# Characterization of Gene Isoforms Related to Cellulose and Lignin Biosynthesis in Kenaf (*Hibiscus cannabinus* L.) Mutant

**DOI:** 10.3390/plants9050631

**Published:** 2020-05-14

**Authors:** Jae Il Lyu, Rahul Ramekar, Dong-Gun Kim, Jung Min Kim, Min-Kyu Lee, Nguyen Ngoc Hung, Jin-Baek Kim, Joon-Woo Ahn, Si-Yong Kang, Ik-Young Choi, Kyoung-Cheul Park, Soon-Jae Kwon

**Affiliations:** 1 Advanced Radiation Technology Institute, Korea Atomic Energy Research Institute, Jeongeup 56212, Korea; jaeil@kaeri.re.kr (J.I.L.); dgkim@kaeri.re.kr (D.-G.K.); jmkim0803@kaeri.re.kr (J.M.K.); biolmk@kaeri.re.kr (M.-K.L.); nguyenhung@kaeri.re.kr (N.N.H.); jbkim74@kaeri.re.kr (J.-B.K.); joon@kaeri.re.kr (J.-W.A.); sykang@kaeri.re.kr (S.-Y.K.); 2 Department of Agriculture and Life Industry, Kangwon National University, Chuncheon 24341, Korea; Rahul.ramekar@gmail.com (R.R.); choii@kangwon.ac.kr (I.-Y.C.)

**Keywords:** gamma ray, mutant, kenaf, iso-seq, cellulose, lignin

## Abstract

Kenaf is a source of fiber and a bioenergy crop that is considered to be a third world crop. Recently, a new kenaf cultivar, "Jangdae," was developed by gamma irradiation. It exhibited distinguishable characteristics such as higher biomass, higher seed yield, and earlier flowering than the wild type. We sequenced and analyzed the transcriptome of apical leaf and stem using Pacific Biosciences single-molecule long-read isoform sequencing platform. De novo assembly yielded 26,822 full-length transcripts with a total length of 59 Mbp. Sequence similarity against protein sequence allowed the functional annotation of 11,370 unigenes. Among them, 10,100 unigenes were assigned gene ontology terms, the majority of which were associated with the metabolic and cellular process. The Kyoto encyclopedia of genes and genomes (KEGG) analysis mapped 8875 of the annotated unigenes to 149 metabolic pathways. We also identified the majority of putative genes involved in cellulose and lignin-biosynthesis. We further evaluated the expression pattern in eight gene families involved in lignin-biosynthesis at different growth stages. In this study, appropriate biotechnological approaches using the information obtained for these putative genes will help to modify the desirable content traits in mutants. The transcriptome data can be used as a reference dataset and provide a resource for molecular genetic studies in kenaf.

## 1. Introduction

Kenaf (*Hibiscus cannabinus* L.) is an annual, warm season, C3 fiber crop of family Malvaceae native to Africa and Asia. Kenaf has been cultivated since ancient times (4000 years ago) and traditionally was used to make ropes, canvases, and sacks [1,2]. Furthermore, the global demand for fibrous material and the worldwide shortage of trees have shifted the focus to nonwood crops such as kenaf as a source of natural and biodegradable fibers [3]. Kenaf also has a gained attention as a bioenergy crop. Within a short time period (6 months), kenaf can grow taller than southern pine trees to heights of 2–4 m, which contributes to its high biomass yield [4]. Kenaf has a high photosynthesis rate and high ability to absorb atmospheric CO_2_, thereby benefiting the ecosystem [5]. It is probably the most important fiber plant after cotton and jute, yet it is still considered as a third world crop. This is partly because of the lack of genomic information and genetic tools for kenaf, which has restricted breeding programs to accelerate genetic improvements in this plant.

Kenaf cultivars can be divided into three maturation groups based on flowering time: early, mid-late, and late maturing. Early-maturing cultivars produce less biomass but have high seed yield; late maturing cultivars produce more biomass but with the risk of seed shattering and reduced seed quality; and mid-late cultivars balance high biomass production with good seed quality and quantity [6,7]. Recently, a mid-late cultivar, “Jangdae”, was developed by mutation breeding using gamma irradiation [8]. Compared with early and late maturing cultivars, Jangdae exhibited the desirable characteristics of high biomass and high seed yield, making it an attractive model plant for animal feed and industrial materials. However, there is still scope to improve the fiber quality of Jangdae, which is not significantly different from that of other cultivars. An understanding of the mechanism of Jangdae fiber development would be valuable for further genetic engineering and molecular breeding of this cultivar.

Transcriptome analysis based on next-generation sequencing (NGS) is used to achieve a dynamic range of gene expression levels and provide a broad understanding of transcriptional and post-transcriptional gene regulation [9] in fiber crops such as cotton [10,11], jute [12], and kenaf [13,14,15]. Although the short reads generated by NGS are a powerful resource, they do not span the full length of most transcripts, making it challenging to annotate and characterize genes, especially isoforms, and to perform de novo assemblies [16]. The single-molecule real-time (SMRT) sequencing technology developed by Pacific Bioscience (PacBio) can produce long length reads for transcriptome data. SMRT sequencing together with isoform sequencing (Iso-Seq) is used to generate and characterize long read transcripts with a low error rate [17]. Unlike other NGS techniques that require a PCR amplification step before sequencing, SMRT techniques omit the amplification step, thus preventing PCR bias errors. Long length reads also facilitate de novo assembly, which is difficult using short length reads where repeat elements, structural errors, incomplete assembly, and base error can lead to unreliable gene annotation. Long read length reads can deliver complex de novo genome assemblies with fully or partially closed gaps [17,18]. The PacBio SMRT iso-seq technology was used effectively for transcriptome data analysis in major crops, including maize [19], wheat [20] and sorghum [21].

In this study, we aimed to improve the accuracy of genetic prediction in the Jangdae cultivar, using the SMRT iso-seq protocol to generate full-length or partially assembled transcripts followed by de novo assembly and characterization. Our objectives were: (1) to characterize and functionally annotate full-length transcripts with a broad survey of genes associated with various biological process; and (2) to identify fiber-related genes involved in cellulose and lignin biosynthesis, and further check the expression pattern of few selected genes involved in lignin biosynthesis at different growth stages.

## 2. Results

### 2.1. Transcriptome Assembly and Gene Annotation

**Figure 1:**
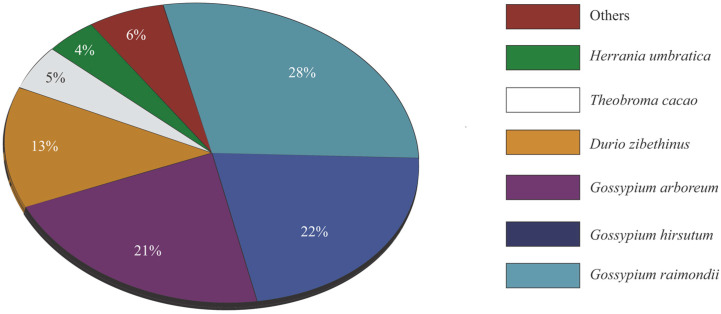
Distribution of top-hit species in BLASTx searches of kenaf unigenes against the NCBI nr and UniProt databases.

We sequenced the transcriptome of kenaf and after clustering and polishing obtained 26,822 high-quality full-length consensus transcripts with a total length of 59,000,000 base pairs. Subsequently, 19,775 nonredundant representative sequences with total length of 43,000,000 base pairs were filtered using CD-HIT, and a final set of 11,370 unigenes with total length of 16,000,000 base pairs (Table 1 and Table S2) were further processed for gene annotation. BLASTx analysis was performed against the NCBI nr and UniProt databases and 11,291 (99%) of the unigenes had at least one positive BLAST hit. Distribution analysis indicated that the sequences of six species had hits with more than 450 transcripts. In particular, 61% of the unigenes shared high homology with three *Gossypium* species from family Malvaceae, namely, *Gossypium raimondii* (28%, 3201 reads), *Gossypium hirsutum* (22%, 2507 reads), and *Gossypium arboreum* (21%, 2441 reads), which indicates they are phylogenetically closely related (Figure 1).

### 2.2. Functional Annotation, KEGG Classification, and Isoform Analysis

**Figure 2:**
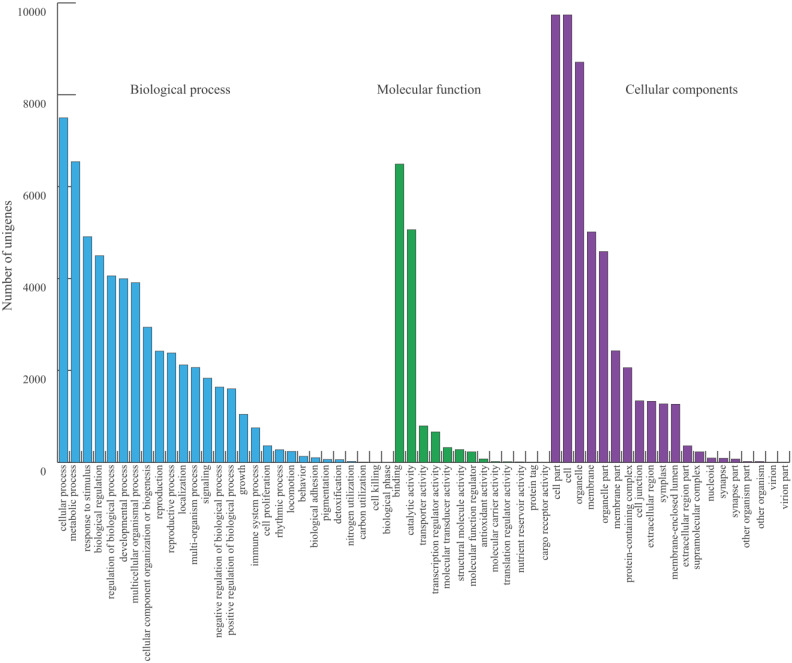
Enrichment analysis of the gene ontology terms assigned to the final set of kenaf unigenes.

To analyze the functions of the genes and protein products, we assigned GO terms to the final set of 11,370 unigenes. In total, 10,100 (88.8%) transcripts were assigned to 61 functional groups under the three main categories: cellular components (9864, 97.66%), biological process (8647, 85.61%), and molecular function (8378, 82.95%). Under biological process, “cellular process” and “metabolic process” were the most represented terms; under molecular function, “binding” and “catalytic activity” were the most represented; and under cellular component, “cell part” and “cell” were the most represented (Figure 2 and Table S3).

**Figure 3:**
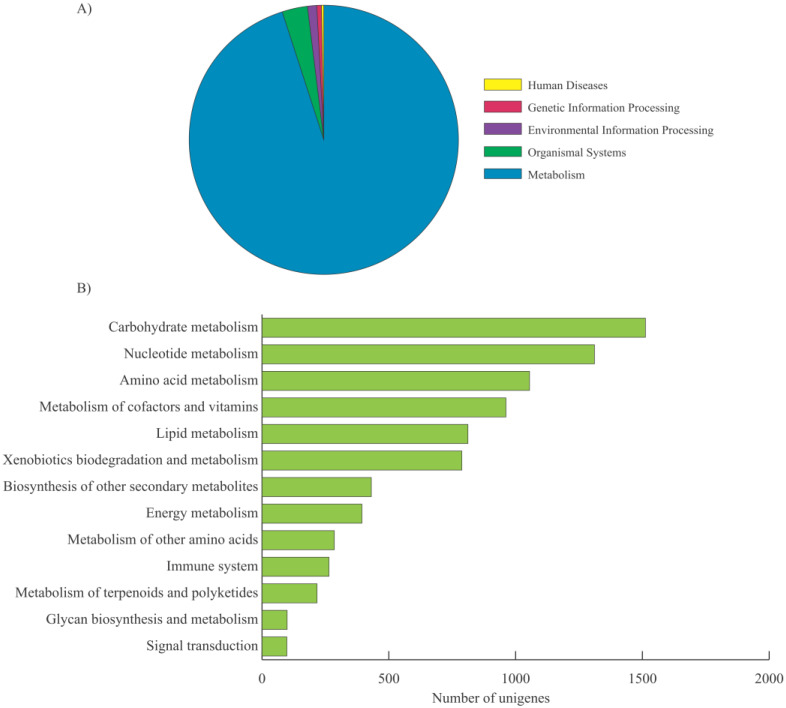
Enrichment analysis of the KEGG pathways assigned to the kenaf unigenes. (**A**) Distribution of the kenaf unigenes in KEGG biological categories. (**B**) Classification of the kenaf unigenes under “metabolism”.

The unigenes with assigned GO terms were compared against the Kyoto encyclopedia of genes and genomes (KEGG) database for pathway analysis. In total, 8875 kenaf unigenes were assigned to 149 KEGG pathways and given 813 EC numbers that were used to represent putatively identified genes involved in various pathways. The annotated unigenes were grouped into five major categories (Figure 3A), “metabolism” (8453 unigenes), “organismal system” (264 unigenes), “environmental information processing” (98 unigenes), “genetic information processing” (59 unigenes), and “human disease” (1isoform). Under “metabolism,” the highest number of unigenes were mapped to “carbohydrate metabolism” (1512 unigenes) followed by "nucleotide metabolism” (1311 unigenes), “amino acid metabolism” (1055 unigenes), “metabolism of cofactors and vitamins” (952 unigenes), and “lipid metabolism” (812 unigenes) (Figure 3B). Details of the KEGG analysis, including information for pathways, enzymes in pathways, enzyme annotation, sequences, and categories and subcategories are provided in Table S4. These annotations will provide a valuable resource for further gene function and pathway research.

**Figure 4:**
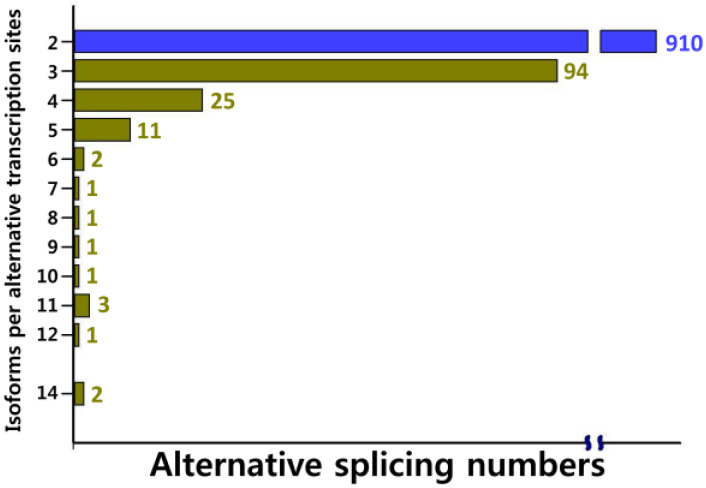
Number of isoforms identified in the alternatively spliced kenaf unigenes.

We successfully characterized and assigned full-length cDNA with isoform information. We detected alternative splicing in 1052 transcripts covering 2376 isoforms with 2–11 isoforms per transcript derived from alternative transcription sites, alternative polyadenylation, or alternative splicing events (Figure 4). The transcript lengths, including the unigenes, were 378–7466 base pairs. Our annotation data showed that 281 clusters containing 628 isoforms were involved in different metabolic pathways.

### 2.3. Identification of Major Genes Involved in Cellulose and Lignin Biosynthesis

#### 2.3.1. Cellulose Biosynthesis

**Figure 5:**
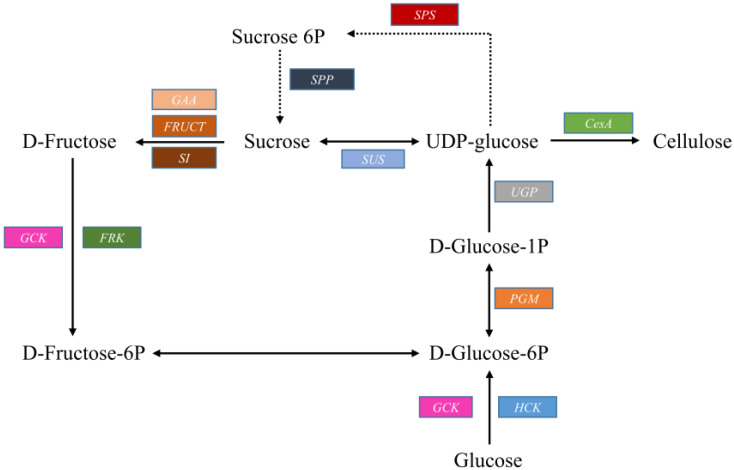
Hypothetical pathway constructed with kenaf unigenes (highlighted) involved in cellulose biosynthesis.

Cellulose synthase (*CesA*) uses UDP-glucose as a direct precursor for cellulose biosynthesis and is a key enzyme involved in the generation of plant cell wall cellulose. UDP-glucose is synthesized from glucose by the cytosoluble enzymes hexokinase (*HK*), phosphoglucomutase (*PGM*), and UDP-glucose pyrophosphorylase (*UGP*). Sucrose synthase, which can synthesize UDP-glucose, is also considered to be involved in cellulose biosynthesis. We detected candidate genes encoding these major enzymes among the annotated unigenes (Table 2). Besides these genes, we also detected genes that encode enzymes that are indirectly (but can be essential) involved in cellulose syntheses, including sucrose-phosphate synthase (*SPS*), sucrose-6-phosphatase (*SPP*), alpha-glucosidase (*GAA*), beta-fructofuranosidase (*FRUCT*), sucrase-isomaltase (*SI*), and fructokinase (*FRK*). Based on previous studies [22,23], a hypothetical pathway was constructed (Figure 5). A total of 109 unigenes coding 12 key enzymes that control the pathway route for cellulose synthesis were identified. Among them, 21 unigenes (most represented) were annotated as encoding CesA subunits.

#### 2.3.2. Lignin Biosynthesis

**Figure 6:**
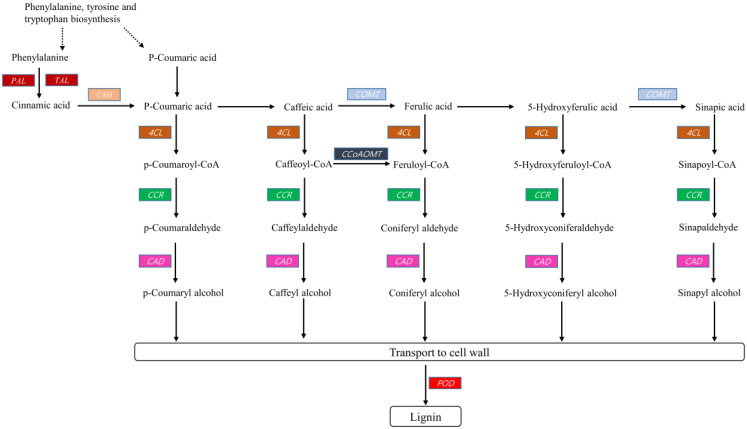
Hypothetical pathway constructed with kenaf unigenes (highlighted) involved in lignin biosynthesis.

Lignin is composed of cross-linked polymers derived from phenolic alcohol or monolignols (sinapyl, coniferyl, and p-coumaryl alcohol). These monolignols are produced in the cytoplasm and transported to the cell walls where they are polymerized by peroxidase or laccases to form lignin. Several genes expressed mainly in xylem parenchyma cells, namely, those encoding phenylalanine ammonia-lyase, tyrosine ammonia-lyase, cinnamate 4-hydroxylase, 4-coumarate CoA ligase, cinnamoyl CoA reductase, p-hydroxycinnamoyl CoA shikimate, p-coumarate 3-hydroxylase (*C3H*), caffeoyl CoA O-methyltransferase, ferulate 5-hydroxylase (*F5H*), caffeoyl shikimate esterase (*CSE*), caffeic acid O-methyltransferase, and cinnamyl alcohol dehydrogenase (*CAD*) are involved in lignin biosynthesis [24]. All the important genes, except *C3H*, *F5H*, and *CSE*, were identified in our data (Table 3). The currently accepted lignin biosynthetic pathway is illustrated in Figure 6. A total of 78 unigenes coding ten key enzymes that control the pathway route for lignin synthesis were identified in our transcriptome data. Among them, 41 unigenes (most represented) encoding peroxidase, a key enzyme, were detected.

### 2.4. Expression Analysis of Lignin Biosynthesis Genes

**Figure 7:**
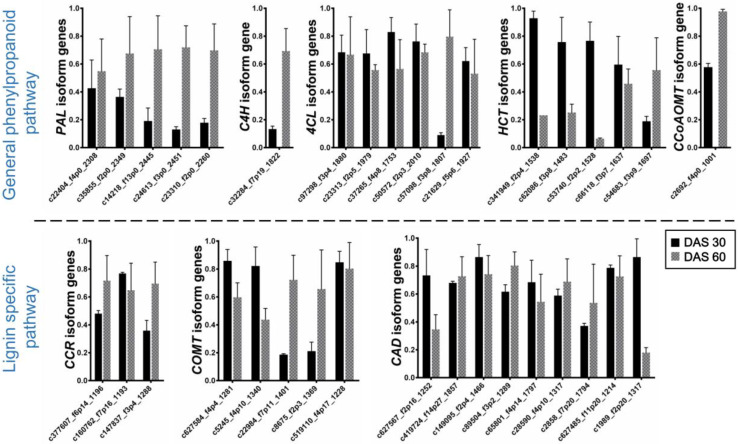
Relative expression time course at 30 and 60 days after seeding of eight genes involved in lignin biosynthesis ascertained by qPCR. Primers were designed on the respective gene sequences (deposited in NCBI database) and used as reference to compare the expression of the current data.

To validate the transcriptome data, we analyzed the expression of eight genes (and respective unigenes) involved in lignin synthesis (*PAL*, *C4H*, *4CL*, *CCR*, *CoAOMT*, *CAD*, *HCT*, and *COMT*) using qPCR (Figure 7).

#### 2.4.1. General Phenylpropanoid Pathway

*PAL* is found as a tetramer in vascular plants and plays a key role in regulating the biosynthesis of phenylpropanoid, including lignin. In our data, we identified five unigenes with sequence length ranging from 2260 bp to 2451 bp. The results showed that *PAL* unigenes exhibited different expression patterns at different growth stages. The expression level at 60 days after seeding (DAS) was much higher, indicating that *PAL* plays an important role at a later stage of plant growth. *C4H* is a cytochrome P-450 linked monooxygenase and catalyzes the first hydroxylation of cinnamic acid in the lignin biosynthesis. *CoAOMT*, on the other hand, plays a role in methylation of both caffeyl-CoA and 5-hydroxyferuloyl-COA during monolignols biosynthesis. We identified only one unigenes corresponding to each unigenes with a sequence length of 822 and 1001, respectively. The expression level for both the unigenes was much higher at 60 DAS compared to 30 DAS.

*4CL* catalyzes the formation of thioesters of cinnamic acids and plays a regulatory role in the biosynthesis of various phenolic derivatives. We identified six unigenes with sequence length varying from 1753 to 2010 bp. No significant differences in expression level between the 30 and 60 DAS stages were observed except in unigene c57098-f3p8-1807, which showed higher expression at 60 DAS. *HCT* is a key metabolic entry point for the synthesis of monomers coniferyl and sinapyl alcohols. We identified five unigenes with sequence length ranging from 1483 to 1697 bp. We observed a clear trend of high expression level at 30 DAS stage without one unigenes (c54683_f3p9_1697), indicating the activity of the gene at an early stage of plant growth for lignin biosynthesis.

#### 2.4.2. Lignin Specific Pathway

*CCR* catalyzes the reduction of hydroxycinnamoyl-COA thioesters to aldehydes and plays a key role in the production of monolignols from phenylpropanoid metabolite, the first step for lignin biosynthesis. We identified five unigenes with sequence length ranging from 1193 bp to 1288 bp. Two out of three unigenes showed slightly higher expression in 60 DAS. *CAD* catalyzes the reduction of hydroxycinnamaldehyde to hydroxyl cinnamyl alcohol dehydrogenase and plays an important role at the end of monolignols biosynthesis. We identified nine unigenes with sequence lengths varying from 1214 to 1857 bp. Similar to *4CL*, no significant difference in expression level between the growth stages were observed. However, unigene c1989_f2p20_1317 exhibited a clear difference in expression level, which was much higher at 30 DAS stage. *COMT* catalyzes the methylation of hydroxyl moiety of monolignols in the S subunit of lignin. We identified five unigenes with a sequence length ranging from 1228 to 1401. Although *COMT* unigenes exhibited different expression patterns in the growth stage, a clear trend was not set as two unigenes showed higher expression for 30 DAS and two unigenes for 60 DAS.

Overall, the genes showed relative expression; however, different expression levels were observed at the various growth stages. Briefly, we could categorize genes into two parts: (a) genes with no significant difference in expression level between the growth stages (*4CL* and *CAD*) and (b) genes exhibiting significant expression pattern between the growth stages (*PAL*, *C4H*, *CCR*, *CoAOMT*, *HCT*, and *COMT*). Four out of eight genes, including *PAL*, *C4H*, *CCoAOMT*, and *CCR*, expressed highly in the latter part of the growth stage, and only one gene (*HCT*) exhibited a clear trend of early expression for lignin biosynthesis.

## 3. Discussion

To accelerate functional genomics research on the elite mutant cultivar Jangdae, we performed de novo transcriptome analysis of young leaf tissue employing a PacBio iso-seq technique. Transcriptome data generated by SMRT iso-seq produces long reads with low error rates because the reads are in consensus from multiple sequencing passes of circular cDNA in the SMRT cells [17,18]. For nonmodel crops like kenaf that lack a reference genome, construction of the transcriptome by de novo assembly using NGS technology is a suitable approach for identifying desirable genes and splicing isoforms [25]. To date, most of the kenaf transcriptome studies were based on short reads generated by NGS technology, preventing the accurate assembly of full-length transcripts [13,14,15].

Our transcriptome data included 26,822 high-quality consensus sequences with a total of 59 Mb length (Table 1). This genome-wide coverage of transcripts with complete open reading frames will act as a reference for future studies, including the development of molecular markers for marker-assisted selection for desirable traits in kenaf breeding. The BLAST searches against the protein sequence databases allowed the successful annotation of 42% (11370) transcripts with high e-values. The number of BLAST hits is low compared with other crops analyzed using the same sequencing strategy [26,27]; nevertheless, the data were sufficient to extract enough useful information to meet the objectives of our study. The low number of BLAST hits can be explained by the limited amount of genomic information available for related species, as well as the different combinations of plant tissues used in previous transcriptome analysis studies. The results of the BLAST searches revealed a close relationship between kenaf and *Gossypium* species (*Gossypium raimondii*, *Gossypium hirsutum*, and *Gossypium arboreum*), in agreement with previous reported results [28,29].

The GO functional classification annotated 10,100 high-quality unigenes, a substantial proportion of which are involved a cellular and metabolic processes with predominant binding and catalytic activity in cells or cellular parts (Figure 2). The annotations provide a foundation to understand genetic networks in plant growth and development, gene regulation, and stress response for kenaf. Further, 8875 of the GO annotated unigenes were assigned to 149 KEGG pathways (Figure 3). More than 95% of the transcripts were found to be involved in metabolic processes, confirming the advantage of full-length transcripts for discovering candidate genes involved in various biosynthesis pathways. Overall, the results confirm the accuracy of our data and provide a foundation to identify genetic networks for desirable traits in kenaf.

Kenaf fiber is characterized chemically by cellulose (58%–63%) and lignin (12%–14%), which are also traits that are important for improving fiber quality [30,31,32]. However, cellulose content in kenaf is relatively low compared with other fiber crops such as flax (78%–80%), hemp (75%–80%), jute (60%–65%), and ramie (70%–75%) [33]. Cellulose is a polymer of β-(1→4)-glucose residues that form a linear unbranched chain. Cellulose forms the structural component of plant cell walls, providing the mechanical support that allows them to stand upright. Lignin, although weaker than cellulose, provides additional tensile strength and contributes to the dry weight of a plant. Lignin improves water conductivity properties and also plays an essential role in plant defense against pathogen attack [34,35]. We examined in detail the enzymes involved in the KEGG starch and sucrose metabolism pathway (related to cellulose biosynthesis) and KEGG phenylpropanoid pathway (lignin biosynthesis). Genetic improvement by raising cellulose content using genetic engineering techniques or mutation breeding will add to the economic value of kenaf. Conversely, high lignin content in fiber crops has a negative effect on forage quality and is a major technical obstacle for the paper industry and bioethanol production [36]; thus, kenaf breeding efforts are aimed at developing cultivars with altered lignin content. We detected most of the genes involved in cellulose (Table 2) and lignin (Table 3) biosynthesis in our transcriptome data. These genes are likely candidates for genetic manipulation for improving or altering the content of these compounds in kenaf. Additional to knowing the genes, the time course of its expression can help to apply modern genomic tools and improve our target trait. Expression pattern analysis of selected genes for lignin biosynthesis from our data showed a change of expression at the different growth stages. Studies showed that fiber production in kenaf peaks at a later stage of plant growth [7,37]. Also, our previous kenaf study reported that lignin content was accumulated approximately four fold at 60 DAS compared with 30 DAS, and exhibited the same tendency in gene expression level of lignin biosynthesis [38]. In the present study, four out of eight isoform genes exhibited a clear trend of high expression at 60 DAS, leading us to suppose that the lignin amount increased by time and growth of the plant. A direct correlation between lignin gene expression and time of plant growth was reported in earlier studies and is consistent with our results [39,40,41]. Interestingly, the expression pattern of *HCT* gene in our data showed a reverse trend, i.e., the gene expressed highly at an earlier growth stage, which is worthy of doing in-depth research. The identified unique unigenes with high expression at a particular growth stage in our data will facilitate the dissection of the molecular and genetic basis of lignin biosynthesis. This approach for applying new genomic technologies will help the discovery of novel genes in mutation breeding and genomic study.

## 4. Materials and Methods

### 4.1. Plant Material and RNA Extraction

The kenaf mutant cultivar “Jangdae” was planted in the experimental field at the Korea Atomic Energy Research Institute (KAERI, Jeongeup, Korea) in 2018. Young apical stem and leaves were collected, immediately frozen in liquid nitrogen, and ground into fine powder. Total RNA was extracted using a Hybrid-RTM kit (GeneAll Biotechnology Co., Seoul, Korea) according to the manufacturer’s protocol. The total RNA quality was assessed using an Agilent 2100 Biosystem (Agilent Technologies Inc., Santa Clara, CA, USA). RNA with a concentration of 1–10 μg and RNA integrity number (RIN) >8.0 was used for sequencing.

### 4.2. PacBio SMRT Iso-Seq Sequencing and Data Analysis

Using a Clontech SMARTer PCR cDNA synthesis kit (Takara Bio USA; Inc., Mountain View, CA, USA), cDNA was synthesized from the extracted RNA, followed by size selection (1–6 kb) using a Bluepippin^TM^ system (Sage Science Inc., Beverly, MA, USA). A template library was prepared using a SMRTbell library kit for sequencing on the PacBio RS II platform (Pacific Biosciences, Palo Alto, CA, USA) at the National Instrumentation Center for Environmental Management (NICEM), Seoul National University, South Korea. Raw data were processed following the ToFU (transcript isoform full-length and unassembled) pipeline (GitHub, Pacific Biosciences of California, Inc., Menlo Park, CA, USA). Raw reads were characterized into full-length and non-full-length reads based on primer and poly (A) tail detection. The full-length reads were clustered to predict consensus isoforms, then reclustered with non-full-length reads using Quiver (included in ToFU pipeline) to generate high-quality polished consensus transcripts with an accuracy >99% [42]. Redundant sequences were removed, and orthologous genes were analyzed by clustering the final consensus transcripts using CD-HIT-EST in the CD-HIT package (v.4.6) with a threshold value of 0.99 identity (Li Lab at UCSD, La Jolla, CA, USA). The PacBio-seq raw reads can be accessed at NCBI with the following accession number: SRR8586247 (Sequence Read Archive number; https://www.ncbi.nlm.nih.gov/sra/).

### 4.3. Functional Annotation and Classification

For functional annotation the assembled isoforms were searched against the UniProt and NCBI nonredundant (nr) protein databases using BLASTX with an e-value cutoff of 10^−6^. GO terms and KEGG pathway analyses were performed using Blast2GO [43] against the same two databases. The retrieved GO terms were classified into the three main GO categories, biological process, cell component, and molecular function. Based on similarity hits against the KEGG database, unique enzyme commission (EC) numbers were assigned to transcripts that were then mapped to KEGG biochemical pathways.

### 4.4. Isoform Grouping

Isoform grouping was carried out using the pipeline-to-isoform system of full-length cDNA sequences developed as previously reported [42]. The nonredundant high-quality transcripts that aligned to the longest orthologous consensus reads were used as the reference sequences to identify alternatively spliced isoforms. This procedure was performed in triplicate followed by reclustering with amino acid peptide sequences. The resulting isoforms were validated by alignment with the corresponding consensus transcript sequence using GMAP and by filtering redundant transcripts using TOFU pipelines.

### 4.5. Quantitative PCR/Expression Analysis of Lignin Biosynthesis Genes

Unigenes obtained from data analysis were further validated by qRT-PCR. Eight unigenes involved in lignin biosynthesis were selected for quantitative real-time expression. To evaluate gene expression, RNA was isolated from apical stem and leaves collected from plant tissue at 30 and 90 DAS. Total RNA was isolated using the Trizol reagent with 2-mercaptoethanol. RNA was further reverse transcribed, and first-strand cDNA synthesis was performed on 1 µg of total RNA using the SuperScript III First-Strand Synthesis SuperMix (Invitrogen, Carlsbad, CA, USA). Transcript analysis was performed through real-time qPCR in the Bio-Rad CFX96 Real-Time PCR System (Bio-Rad, Hercules, CA, USA) using SYBR Green SuperMix Kit (Bio-Rad, Hercules, CA, USA). Amplification was carried out through an initial step of 50 °C for 2 min, a denaturation step at 94 °C for 10 min, and 40 cycles of denaturation at 94 °C for 10 s, and annealing and extension at 60 °C for 15 s and 72 °C for 30 s, respectively. Primer pairs (Supplementary Table S1) were designed by using the Primer3 software (http://frodo.wi.mit.edu/primer3/). Expression levels were analyzed with the Eco software (ver. 3.0.16.0) and normalized versus kenaf *ACT7*. Relative values of expression were determined against the maximum value of individual samples at different stages. The reaction was performed in three replicates for each set of conditions and the data presented as means ± SDs (*n* = 3).

## 5. Conclusions

We have characterized the transcriptome of a new kenaf mutant cultivar and broadly surveyed the genetic network involved in various biological processes. We also identified candidate genes involved in cellulose and lignin biosynthesis. The application of appropriate biotechnology tools and approaches will help to reveal information about these genes so that they can be used to modify the content of kenaf plants with the aim of adding economic value to the cultivar. The data will provide an additional reference for future functional and comparative genomics, and our results demonstrate the advantages of SMTR iso-seq data for gene discovery in nonmodel plants.

## Figures and Tables

**Table 1:** Summary of platforms used establishing gene set in kenaf.

Step	Data	Platform	Number of Sequence
1	High quality consensus sequence	RS_IsoSeq	26,822
2	Nonredundant representative sequence	CD-HIT	19,775
3	Reference isoforms	BLASTCLUST and TransDecoder	15,637
4	Final isoforms transcriptome	GMAP and ToFU	12,694
5	Final gene set with representative isoforms	TransDecoder	11,370

**Table 2:** List of candidate genes comprising cellulose biosynthesis pathways found among kenaf unigenes.

Putative Gene	Enzyme	KEGG Ortholog	Enzyme Code	Unigenes
*HK*	Hexokinase	K00844	EC:2.7.1.1	8
*GCK*	Glucokinase	K00845	EC 2.7.1.2	4
*PGM*	Phosphoglucomutase	K01835	EC 5.4.2.2	4
*UGP*	UDP-glucose pyrophosphorylase	K00963	EC:2.7.7.9	5
*CesA*	Cellulose synthase	K10999	EC:2.4.1.12	21
*SUS*	Sucrose synthase	K00695	EC:2.4.1.13	9
*SPS*	Sucrose-phosphate synthase	K07024	EC 2.4.1.14	5
*SPP*	Sucrose-6-phosphatase		EC 3.1.3.24	5
*GAA*	Alpha-glucosidase	K01187	EC 3.2.1.20	19
*FRUCT*	Beta-fructofuranosidase	K01193	EC 3.2.1.26	11
*SI*	Sucrase-isomaltase	K01203	EC 3.2.1.48	11
*FRK*	Fructokinase	K00847	EC:2.7.1.4	7

**Table 3:** List of candidate genes comprising lignin biosynthesis pathways found among kenaf unigenes.

Putative Gene	Enzyme	KEGG Ortholog	Enzyme Code	Unigenes
*PAL*	Phenylalanine ammonia lyase	K10775	EC:4.3.1.24	5
*PAL*	Tyrosine ammonia lyase	K13064	EC:4.3.1.25	5
*C4H*	Cinnamate 4-hydroxylase	K00487	EC:1.14.13.11	1
*4CL*	4-coumarate CoA ligase	K01904	EC:6.2.1.12	6
*CCR*	Cinnamoyl CoA reductase	K09753	EC:1.2.1.44	3
*CAD*	Cinnamyl alcohol dehydrogenase	K00083	EC:1.1.1.195	9
*HCT*	Hydroxycinnamoyl CoA shikimate /quinate phydroxycinnamoyl transferase	K13065	EC:2.3.1.133	5
*CCoAOMT*	Caffeoyl CoA O-methyltransferase	K00588	EC:2.1.1.104	1
*COMT*	Caffeic acid O-methyltransferase	K13066	E2.1.1.68	2
*POD*	Peroxidase	K00430	EC:1.11.1.7	41

## Supplementary Materials

The following are available online at https://www.mdpi.com/2223-7747/9/5/631/s1, Table S1: Primer information used for qPCR analysis, Table S2: Detailed information of the final gene set with representative isoforms, Table S3: GO terms to the final set of 11,370 unigenes. Functional groups under the three main categories: cellular components, biological process, and molecular function, Table S4: Detailed information of the KEGG classification results.
